# Case report of right supernumerary kidneyin a 38 year old male

**DOI:** 10.1016/j.radcr.2024.06.016

**Published:** 2024-07-03

**Authors:** Ahmed Nada, Ash Jhamb, Julian Maingard

**Affiliations:** a St Vincent's Hospital Melbourne, 41 Victoria Parade, Fitzroy VIC 3065, Australia; b St Vincent's Hospital Melbourne, Interventional Radiology Department, 41 Victoria Parade, Fitzroy VIC 3065, Australia

**Keywords:** Supernumerary kidney, Renal artery aneurysm

## Abstract

Supernumerary kidney is a highly uncommon congenital anomaly characterized by the presence of at least 1 or more extra kidneys in variable positions and morphology. Due to the scarcity in the medical literature, not much is known about this condition. The additional kidney typically has its own collecting system, vascular supply, and distinct encapsulated parenchyma. In this case, we present a 38-year-old male with a right supernumerary kidney who was initially investigated for a renal artery aneurysm. In the case of our patient, the discovery of a supernumerary kidney influenced the course of management and prevented unnecessary intervention and procedure. This emphasizes the importance of thorough imaging and diagnostic techniques in patients to accurately identify and characterize such anomalies.

## Introduction

Supernumerary kidney, an exceedingly rare congenital renal anomaly, is characterized by the presence of 1 or, even more infrequently, more than 1 additional kidney. Estimates of its prevalence vary, estimated to occur in 0.04% of the population [1]. Remarkably, the medical literature reports fewer than 100 documented cases of supernumerary kidney in the last 2 decades [2]. The supernumerary kidney has unique attributes, including a distinct capsule, vascular supply, and collecting system [2]. Functionally, it may closely resemble the native kidney.

Supernumerary kidneys have been observed in diverse locations, though with limited data available from previous case. They can be present on the left side, caudal to the native kidney [3]. The additional kidneys can present with various symptoms. Symptoms such as pain, fever, or abdominal mass could be attributed to complications such as hydronephrosis, pyelonephritis, malignant tumors, or calculi. Supernumerary kidneys have also been reported to be associated with various conditions, including ectopic ureteric openings, urethral atresia, and urethral duplication.

This case report underscores the significance of meticulously identifying a supernumerary kidney, as exemplified in this instance and, preventing the patient from undergoing unnecessary procedures and investigations.

## Case presentation

**Fig. 1:**
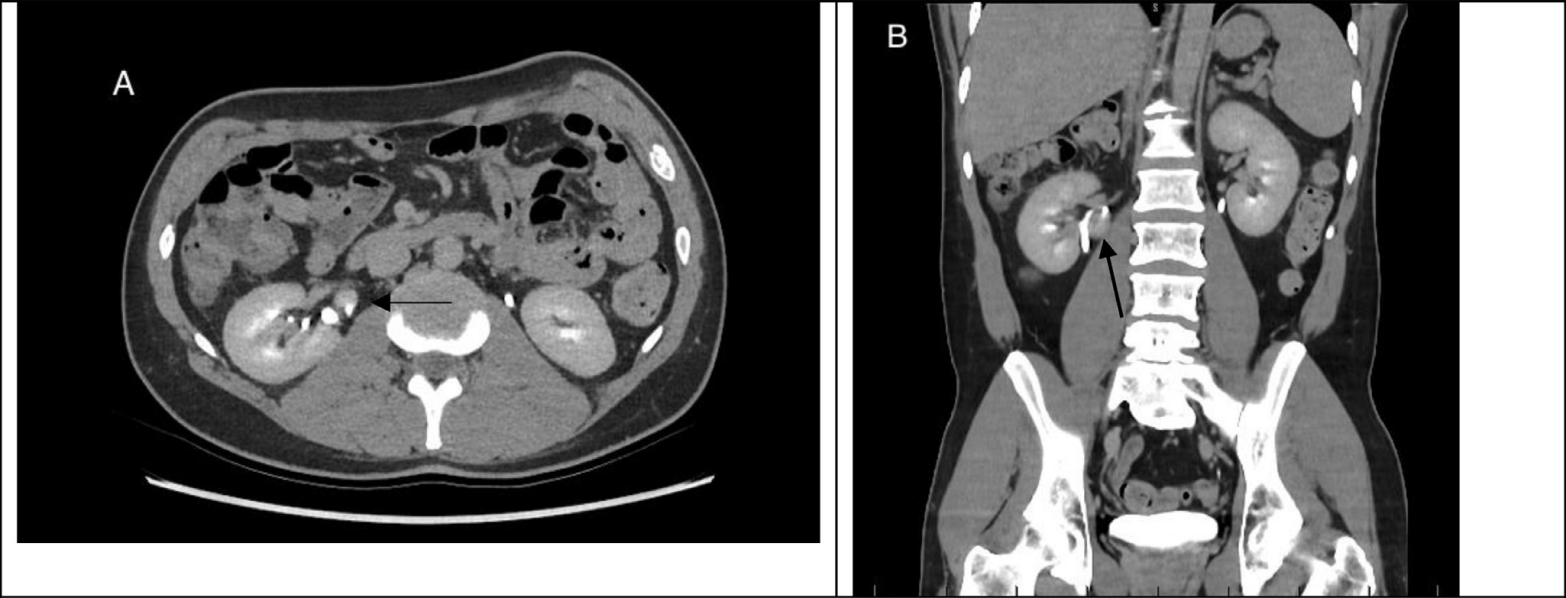
Axial image of computed tomography of the abdomen (A) demonstrating the collecting system (arrow) of the right supernumerary kidney with the upper calyx draining into the main ureter with the right Kidney (B).

A 38-year-old male with a one-month history of intermittent right flank pain presented at a regional emergency department. A computed tomography (CT) scan of the Kidney, Ureter, and Bladder (CT KUB) was performed, revealing a mass around the right kidney. The patient underwent a CT IVP (Intravenous Pyelogram) (Fig. 1) to further characterize the mass. The patient had a medical history including Type 2 diabetes mellitus, dyslipidaemia, and a prior appendectomy. He had a 7 pack year smoking history and consumed alcohol occasionally.

**Fig. 2:**
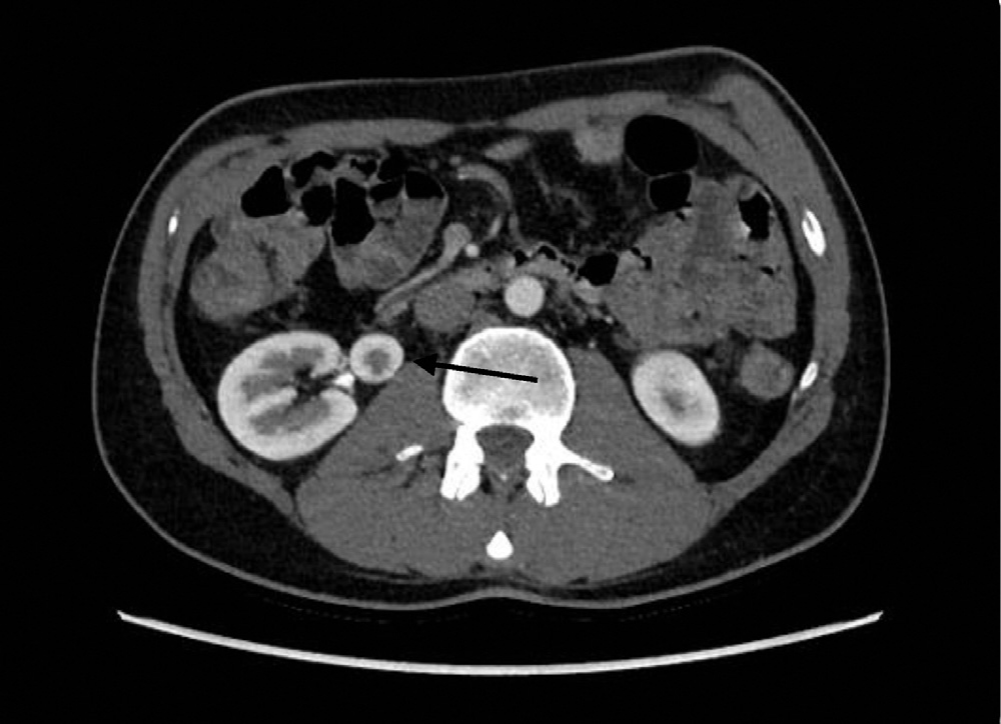
Axial image of computed tomography (CT) in post venous phase showing well defined lesion (arrow) medial to the right kidney. Post contrast enhancement compatible with adjacent kidney and similar morphology. The lesion demonstrates cortical and medullary differentiation as seen on the normal kidney.

CT IVP demonstrated normal collecting systems of left and right kidney however it revealed what was described as an aneurysm of the medial inferior aspect of the right kidney. Consequently, the patient was referred and directly admitted to our hospital for additional investigations including a CT angiogram to further characterize and potentially embolize the aneurysm. After careful review of the CT scans, it became evident that the mass shared morphological features with the parenchyma of the native kidneys (Figs. 1 and 2) and had a collecting system that drained directly into the right kidney's ureter, as seen on the CT IVP (Fig. 1). The findings were consistent with supernumerary kidney rather than renal artery aneurysm (Figs. 1 and 2). Therefore, with the current and new diagnosis the patient was subsequently discharged with no further intervention.

## Discussion

Supernumerary kidney is a rare congenital anomaly and the mechanisms underlying its development are not fully understood, despite various proposed theories. One theory suggests that abnormal division of the nephrogenic cord leads to the formation of 2 metanephric blastemas, resulting in the unilateral development of two separate renal systems, often with a partially or fully segregated ureteral bud [[4], [5], [6]]. Other theories propose that anomalies in the division and migration of the kidney during embryonic development can lead to the formation of an extra kidney [6,7]. The extra kidney has its own separate capsule, collecting system and vascular supply. Supernumerary kidneys occur with equal prevalence among males and females and are often asymptomatic. Thus, they are frequently diagnosed incidentally during investigations for other conditions, as was the case with our patient. They can also report on either side of the abdomen [3].

**Fig. 3:**
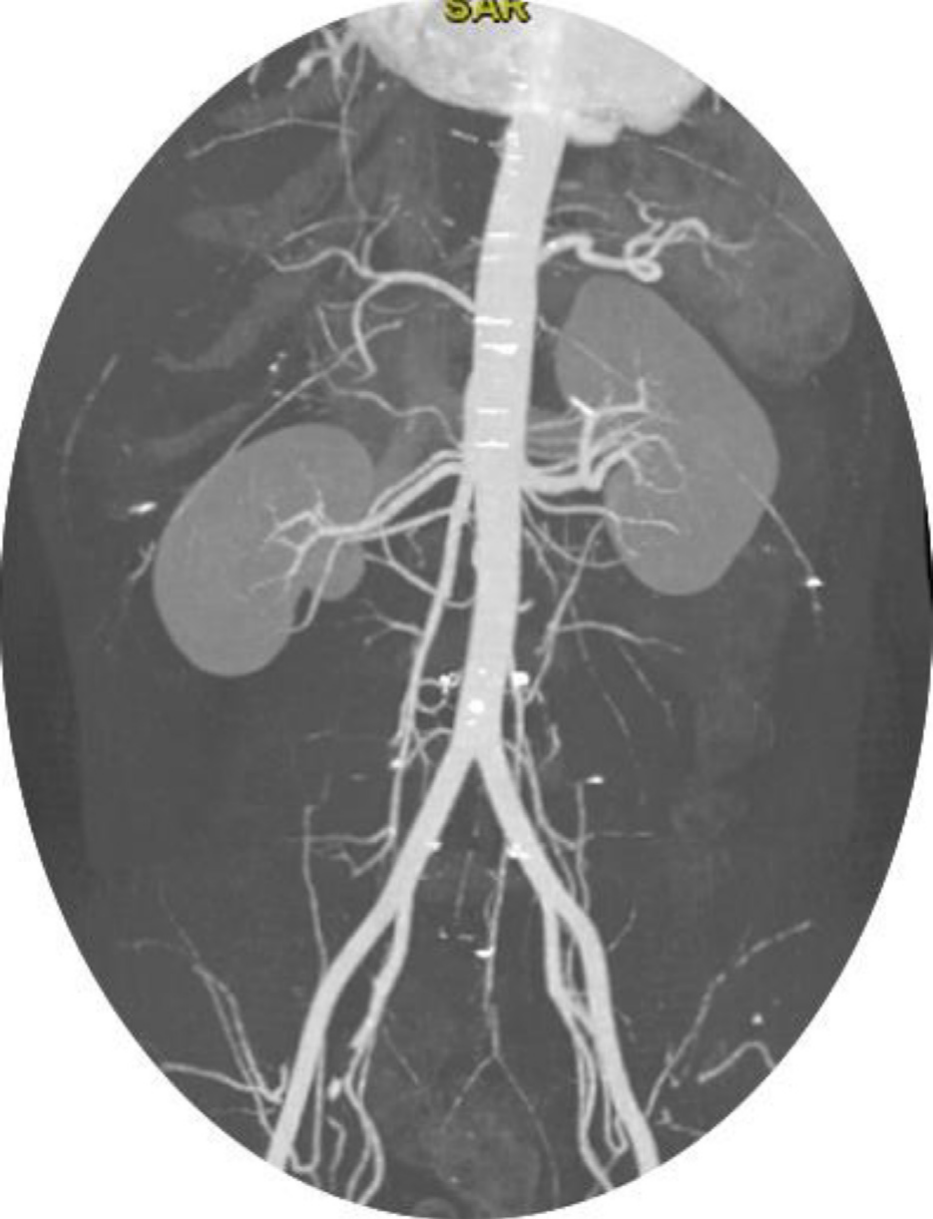
Arterial supply of the SNK, sharing the same arterial supply as the right kidney. Renal artery branching from the abdominal aorta.

The diagnosis of a supernumerary kidney in our 38-year-old male patient underscored the critical role of careful review of radiographic findings. Initially the mass identified on CT scan was suspected to be a renal artery aneurysm based on the location and appearance. However, after a detailed review by the radiology consultant using both the existing CT and CT IVP, which revealed that this mass shared similar morphological features with the parenchyma of the native kidney and possessed a separate collecting system (Figs. 1 and 2). In addition, the CT scans demonstrates that the supernumerary kidney has the same arterial supply by the right renal artery (Fig. 3). This essentially facilitated the correct diagnosis of supernumerary kidney and prevented the patient from undergoing unnecessary vascular intervention.

Supernumerary kidney prevalence is equal between males and females. It is often asymptomatic, as demonstrated in our case. The rarity and often incidental nature of their discovery highlight the need for increased awareness and careful imaging analysis when unusual renal masses are detected.

Accurate diagnosis relies heavily on imaging modalities. In this case, both computed tomography (CT) and CT intravenous pyelogram were instrumental in determining the nature of the mass and distinguishing it from vascular anomalies. No further imaging was needed or performed. The use of dimercaptosuccinic acid (DMSA) scintigraphy is considered valuable for enhanced imaging and functional assessment of supernumerary kidneys (Conrad et al., 1987), contemporary evidence underscores the efficacy of CT scans.

Management of supernumerary kidneys is individualized as demonstrated in this case, lacking established routine guidelines. Asymptomatic cases, incidentally reported, warrant evaluation for concurrent anomalies, and may be observed with interval surveillance. Conversely, in instances of complicated supernumerary kidneys, characterized by complications such as infection, pain, or non-obstructing stones, conservative measures involving antibiotics and analgesia are indicated. In cases of malignancy, consideration should be given to supernumerary nephrectomy [1,8]. The absence of symptoms related to the supernumerary kidney coupled with the incidental history of musculoskeletal pain due to recent physical exertion, guided the decision towards conservative management and surveillance.

## Conclusion

Supernumerary kidneys are very rare congenital anomalies, often asymptomatic but potentially associated with various conditions and complications. Careful investigation and diagnosis are crucial to avoid any unnecessary procedures. Continued reporting of cases involving supernumerary kidneys and other rare congenital anomalies contributes to the ongoing effort to expand our understanding of renal variations and refine clinical practices. This, in turn, may help improve patient outcomes by avoiding unnecessary procedures and ensuring appropriate management strategies for these uncommon conditions.

## Patient consent

It is hereby confirmed that there is an informed consent from the patient referenced in the case report for publication purposes. All identifiable information has been thoroughly removed, ensuring complete anonymity, and preventing any traceability back to the patient. I am committed to upholding the principles of patient confidentiality and privacy throughout the publication process.
